# Rapid Quantification and Quantitation of Alkaloids in Xinjiang *Fritillaria* by Ultra Performance Liquid Chromatography-Quadrupole Time-of-Flight Mass Spectrometry

**DOI:** 10.3390/molecules22050719

**Published:** 2017-05-01

**Authors:** Aziz Mohammat, Abulimiti Yili, Haji Akber Aisa

**Affiliations:** 1 Key Laboratory of Xinjiang Indigenous Medicinal Plants Resource Utilization, Xinjiang Technical Institute of Physics and Chemistry, Chinese Academy of Sciences, Urumqi 830011, China; qabdas@163.com; 2 Xinjiang Technical Institute of Physics and Chemistry, Chinese Academy of Sciences, Urumqi 830011, China; abu@ms.xjb.ac.cn; 3 University of Chinese Academy of Sciences, Beijing 100039, China; 4 Xinjiang Institute of Food and Drug Control, Urumqi 830000, China

**Keywords:** UPLC-QTOF-MS, *Fritillaria*e, qualitative and quantitative analysis, alkaloids

## Abstract

The *Fritillaria* genus, including different kinds of medicinal and edible plants belonging to the Liliaceae family which have the function of treating and relieving a cough and eliminating phlegm, is widely planted in Xinjiang (China). There are few comprehensive studies reporting on the characterization of the chemical constituents of *Fritillaria* from Xinjiang, and to date, no work describing the quantitative differences between the components in *Fritillaria* from Xinjiang and related species. The purpose of this study was to develop qualitative and quantitative analytical methods by Ultra Performance Liquid Chromatography-Quadrupole Time-of-flight Mass Spectrometry (UPLC-QTOF-MS) for the rapid quantification and quantitation of alkaloids in wild and cultivated Xinjiang *Fritillaria*, which could be used in the quality control of medicine based on this natural herb. Using the UPLC-QTOF-MS method, the chemical constituents of Xinjiang *Fritillaria* were identified by fragmentation information and retention behavior, and were compared to reference standards. Furthermore, a quantitative comparision of four major alkaloids in wild and cultivated Xinjiang *Fritillaria* was conducted by determining the content of Sipeimine-3β-d-glucoside, Sipeimine, Peimisine, and Yibeinoside A, respectively. A total of 89 characteristic peaks, including more than 40 alkaloids, were identified in the chromatographic results of *Fritillaria*. Four main alkaloids were quantified by using a validated method based on UPLC-QTOF-MS. The relative contents of Sipeimine-3β-d-glucoside, Sipeimine, Peimisine, and Yibeinoside A varied from 0.0013%~0.1357%, 0.0066%~0.1218%, 0.0033%~0.0437%, and 0.0019%~0.1398%, respectively. A rough separation of wild and cultivated *Fritillaria* could be achieved by the cluster analysis method.

## 1. Introduction

*Fritillaria* represents the bulbs of various plants from the *Fritillaria* genus in the Liliaceae family, which have been used in traditional Chinese medicine for a long period of time because of their effects of clearing heat, moistening the lung, resolving phlegm, and relieving coughs, for the treatment of a cough caused by lung heat and dryness, a low sputum dry cough, a cough due to a yin deficiency, and sputum with blood [1,2,3]. Various bioactive chemical components have been found in *Fritillaria*, mainly consisting of alkaloids, saponins, terpenes, and glycosides. Studies on the chemical constituents and pharmacological actions have shown that the active ingredients resulting in the cough-curing and phlegm-reducing effects were alkaloids [3,4]. Normally, there are five kinds of *Fritillaria*, including *Fritillaria* Cirrhosa, *Fritillaria* ussuriensis, *Fritillaria* pallidiflora, *Fritillaria* thunbergii, and *Fritillaria* hupehensis. Among them, the wild species of *Fritillaria* pallidiflora Schrenk is only distributed in the regions north of the Tianshan Mountains (Xinjiang, China) [5]. With the increasing demand of *Fritillaria* Pallidiflora Schrenk, traditional wild medicine could not satisfy the market demand and cultivated species were introduced. However, whether the quality of cultivated *Fritillaria* Pallidiflora Schrenk possesses a similar effect as the wild medicine has required scientists to discover effective methods for the quality control of the specimens, including the approaches for determining the four main alkaloids in *Fritillaria*: Sipeimine-3β-d-glucoside, Sipeimine, Peimisine, and Yibeinoside A.

Several studies have been reported to determinate the content of alkaloids as the evaluating criteria by adopting methods based on HPLC-UV, HPLC-ELSD, precolumn derivatization HPLC-UV, TLC scanning, spectrophotometry, and LC-MS [6,7,8,9,10,11]. However, it is difficult to directly analyze *Fritillaria* pallidiflora Schrenk by these methods due to the lack of UV chromophores on steroidal alkaloids (for example, classic Sipeimine-3β-d-glucosid, Sipeimine, Peimisine, and Yibeinoside). The treatment of pre-column derivatization HPLC-UV is normally useful, but is very complicated, and HPLC-ELSD suffers from a low sensitivity [12]. With the advantages of a short analysis cycle, strong separating capability, high resolution and sensibility, accurate molecular mass measurement of compounds, and rapid identification of the composition, UPLC/Q-TOF-MS has been widely applied in the study of the material base of natural products, due to its high efficiency in analyzing the chemical components and metabolomics of specimens [13,14,15]. The advantage of a mass spectrometric detector in relation to the selectiveness and sensitivity reveals that it may be effective in analyzing alkaloids in *Fritillaria*. 

In this study, an ultra performance liquid chromatography-quadrupole time-of-flight mass spectrometry (UPLC-Q-TOF-MS) method was established to rapidly analyze the chemical components of *Fritillaria* from both wild and cultivated resources for a detailed comparision, especially for alkaloids with the above reported activities, which provided a reference for medicinal quality control and material basis research. Then, a specific, accurate, and reproducible quantitative method was developed based on UPLC-QTOF-MS, to determine the content of four alkaloids (Sipeimine-3β-d-glucoside, Sipeimine, Peimisine, and Yibeinoside A) in *Fritillaria* from 23 different sources, which is useful for the quality control of *Fritillaria*. The result was analyzed by SPSS (Statistical Product and Service Solutions) 22 software. To the best of our knowledge, no analysis method based on UPLC-QTOF-MS for the quantitative and qualitative determination of alkaloids in *Fritillaria* has been reported.

## 2. Results

### 2.1. Characterization of Chemical Constituents

**Figure 1:**
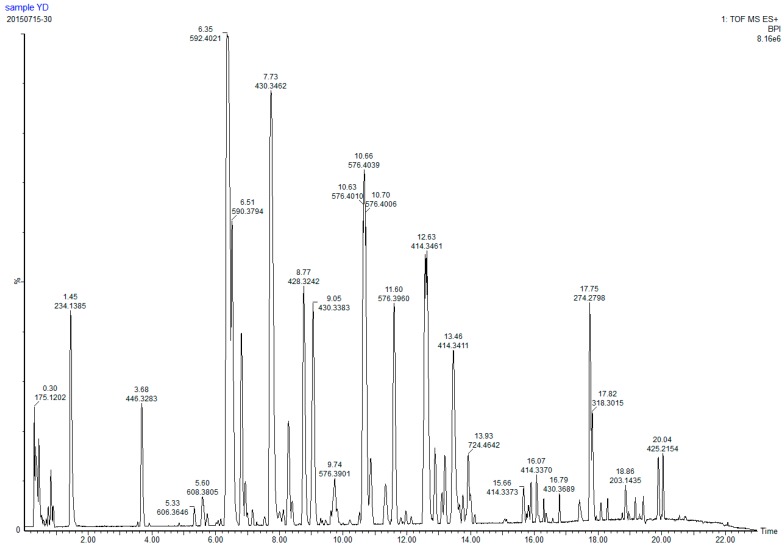
Base peak chromatogram of *Fritillaria* ingredients.

**Figure 2:**
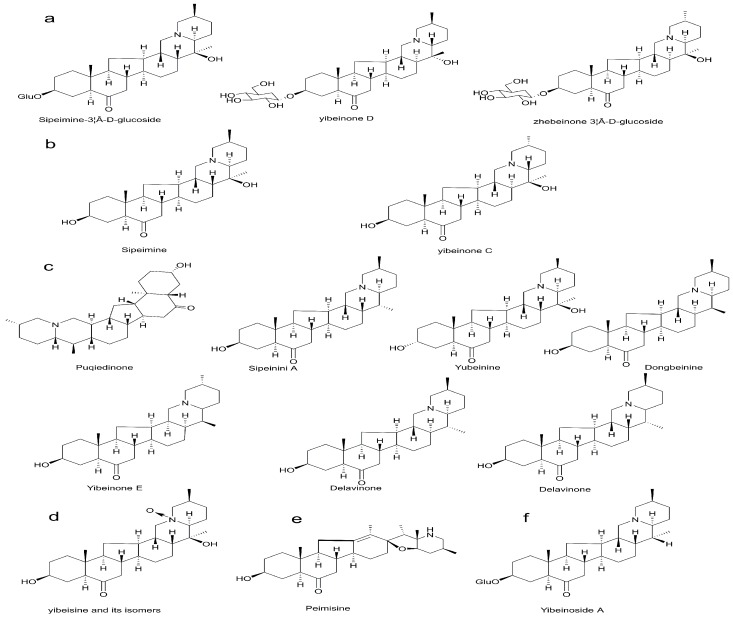
Structure of (**a**) Sipeimine-3β-d-glucoside and its isomers; (**b**) Sipeimineand and its isomers; (**c**) Puqiedinone and its isomers; (**d**) Yibeisine and its isomers; (**e**) Peimisine, and (**f**) Yibeinoside A.

The components of both wild and artificial cultivated *Fritillaria* were analyzed by UPLC-QTOF-MS and relative information is listed in Figure 1. The accurate molecular mass of the positive molecular ion peaks of each chromatogram was obtained, and possible molecular formulas were calculated by MassLynx MS software. A total of 89 and 85 characteristic peaks are elucidated in wild and artifical cultivated *Fritillaria*, respectively, showing that the number of components is similar. By analysing the chromatographic peaks according to the fragment ion peak information and reference data, more than 40 alkaloids were identified (as shown in Table 1 and the structural formula in Figure 2). The value of the mass weight measured by mass spectroscopy is well matched with the value calculated.

### 2.2. Quantitative Analysis

In order to conduct an accurate quantitation of the alkaloids in *Fritillaria*, four alkaloids with a high abundance and good resolution in the chromatogram (sipeimine-3β-d-glucoside, sipeimine, peimisine, and Yibeinoside A) were separated from the 40 compounds and were used as the marker of quantitation. By using the area normalization method, the content of the main ingredients could be ascertained and are listed in Table 2. The ingredients from wild *Fritillaria* with a higher relative mass fraction included Sipeimine-3β-d-glucoside and its isomers (15.61%), Sipeimine-3β-d-glucoside and its isomers (15.58%), puqiedinone and its isomers (14.08%), and Peimisine-3-*O*-β-d-glucopyranoside and its isomers (4.9%). The ingredients from cultivated *Fritillaria* with a higher relative mass fraction included Sipeimine-3β-d-glucoside and its isomers (17.49%), puqiedinone and its isomers (12.85%), Sipeimine-3β-d-glucoside and its isomers (12.71%), Yibeinoside A and its isomers (11.55%), Peimisine-3-*O*-β-d-glucopyranoside and its isomers (6.28%), and Peimisine and its isomers (5.15%), revealing certain differences between wild and cultivated *Fritillaria*. Hence, we chose sipeimine-3β-d-glucoside, sipeimine, peimisine, and Yibeinoside A for the further quantitation of the alkaloids in *Fritillaria*.

#### 2.2.1. Method Validation

Three reference standards (Sipeimine-3β-d-glucoside (99.1%, Batch No.110767-201208, Beijing, China), Sipeimine (96.4%, Batch No.111917-201202, Beijing, China), and Peimisine (≥98%, Batch No. 111892-201402, Beijing, China) were brought from National Institutes for Food and Drug Control, and Standard of Yibeinoside A (>98%, Batch No.141009, Chengdu, China) was used to test the method of validation of the quantitative analysis of alkaloids in *Fritillaria*. Standard curves of sipeimine-3β-d-glucoside, sipeimine, peimisine, and Yibeinoside A were produced with the methods mentioned in Section 4.3.2. As listed in Table S1, all of the regression equations were larger than 0.999, and both the LODs and LOQs were of a nanogram grade, suggesting that the proposed method had a high precision and was available for quantitation. The precision and repeatability of the method built were investigated by continuously analyzing the same sample solution six times and the results are listed in Table S2. The RSD in the precision degree of the four target compounds were 4.89%, 1.26%, 1.37%, and 2.39%, indicating that the precision was very good. The RSD value of the repeatability experiment for the four compounds was satisfactory, with values of 6.53%, 5.26%, 5.14%, and 6.95%, respectively. 

The experiments were relatively stable and reliable for 24 h, because the values of RSD in the stability experiment were all below 6.28%. Five doses of sample 8 were prepared to test the solution and were injected to analyze the contents of the four compounds (see results in Table S2, indicating a good reproducibility of the method). Based on the record in Chinese Pharmacopoeia (2015) (Part 3) [28], the accuracy of the method should be investigated prior to measurement. Hence, standard addition recovery was conducted to investigate the accuracy of the method. The recoveries of the standard solution at low, mediate, and high concentration levels were calculated according to the equivalent compound content of 80%, 100%, and 120%, respectively. As shown in Table S3, the method possessed a good recovery rate, ranging from 99.8% to 103.5%, indicating the satisfactory accuracy of this method. 

#### 2.2.2. Quantitative Investigations

**Figure 3:**
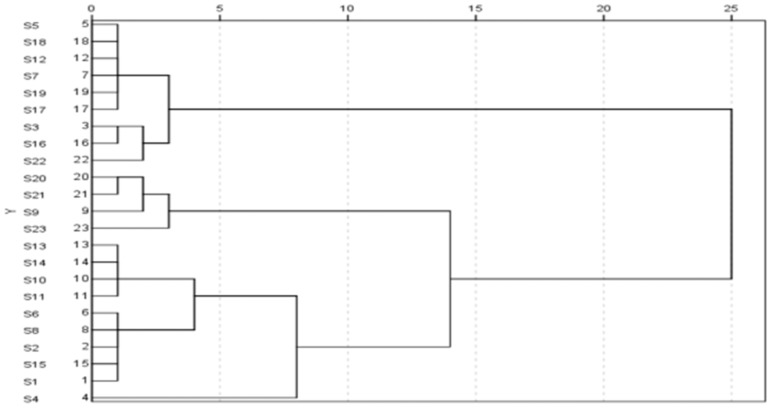
Cluster analysis dendrogram of sample.

The contents of the four alkaloids in 23 samples were analyzed by external standard calibration and the results are presented in Table 2. SPSS 22 software was used as the ward’s method. The measurement range was the square Euclidean distance. The parent sample was analyzed by cluster analysis. In Figure 3, a relationship between the contents of the four alkaloids and sources, as well as the producing areas, can be seen. The differences between the wild and cultivated *Fritillaria* Pallidiflora Schrenk are obvious. S3, S5, S7, S16, S17, S18, S19, and S22 were all wild *Fritillaria* Pallidiflora Schrenk, which could be grouped together. The others could be placed in another group. The content of the four alkaloids in Wild *Fritillaria* S5, S7, S18, S19, and S22 was relatively closer and grew in the Tacheng area. Similarly, the content of the four alkaloids in S20, S21, and S23 was relatively closer and grew in Kazakhstan. The content of the four alkaloids in S1, S2, S4, S10, S11, S13, S14, and S15 was relatively closer, which were wild or cultivated species growing in the Yili area. The cluster analysis results showed the influence of the different area on the medicinal content. 

#### 2.2.3. Producing Area Selection

A total of 23 samples of medicinal *Fritillaria* Pallidiflora Schrenk, including wild and cultivated species, whose plant sources are *Fritillaria*e pallidiflorae bulbus, *Fritillaria*e walujewii, *Fritillaria*e pallidiflora Schrenk, *Fritillaria* tortifolia, Yumin Fritillary Bulb, *Fritillaria* meleagria Linn., *Fritillaria* maximowiczii, *Fritillaria* imperialis, and whose origin include Gongliu County Yili, Nalati Xinyuan County, Yili Tekes County, Toli County Tacheng, Tacheng Yumin County, and Jimunai County Aletai, were included in this investigation. In addition, there three *Fritillaria* samples from Kazakhstan are mentioned in this study. The qualitative result shows that the category of alkaloids found among them is accordance with the results listed in Table 1. Compared with the result of qualification, the quantitation method based on four main alkaloids was more important, which presented the effect of the planting place of *Fritillaria*.

From Kazakhstan, where the climate is similar to Xinjiang, a chemical composition analysis of wild and cultivated species showed the content differences among all kinds of *Fritillaria* from different places. The retention time, molecular ion peak, and relative molecular mass of 23 components of *Fritillaria* were compared with the four alkaloids, which laid a foundation for the quality control and material basis of *Fritillaria*. 

#### 2.2.4. Index Components Selection

Various bioactive constituents have been found in *Fritillaria*, and alkaloids were the active ingredients relative to the cough-curing and phlegm-reducing effects. The alkaloids of *Fritillaria* pallidiflora were normally steroidal alkaloids, like Sipeimine-3β-d-glucoside, Sipeimine, Peimisine, and Yibeinoside A/B. The Chinese Pharmacopoeia of the 2015 Edition, which includes *Fritillaria* pallidiflora, used Sipeimine-3β-d-glucoside and Sipeimine as index components for the content determination of no less than 0.07%. Considering the diversity of chemical components and compounds of a higher relative mass fraction in chemical analysis, this work added Peimisine and Yibeinoside A as index components to increase the quality control level of *Fritillaria* pallidiflora. 

#### 2.2.5. Sample Pretreatment

Alkaloids mostly exist in the plant cells as salt, but only free alkaloids are fat-soluble. Therefore, they must first be alkalized to form their free form by lime milk, sodium carbonate solution, or dilute ammonia water, and then extracted with a lipophilic-organic-solvent like chloroform, dichloromethane, or benzene [27]. Briefly, 0.25 g of *Fritillaria* powder was put into a 50 mL conical flask, and 25 mL of methanol–water (6:4, *v*/*v*) mixture was added. Then, the suspension was sonicated (100 W, 35 kHz) for 30 min. Methanol–water (6:4, *v*/*v*) mixture was then added. After being filtered and centrifugated (5000 rpm), the supernatant was collected and dried, and the residues were re-dissolved in 5 mL methanol prior to analysis. The result of the sample recovery shows that this method had a good extraction efficiency for Sipeimine-3β-d-glucoside, Sipeimine, Yibeinoside A, and Peimisine. 

#### 2.2.6. Result Analysis of the Content Determination

The four alkaloids from the *Fritillaria* samples from 23 sources exhibited large variations, and the relative contents of Sipeimine-3β-d-glucoside, Sipeimine, Peimisine, and Yibeinoside A were 0.0013% (S18)~0.1357% (S23), 0.0066% (S18)~0.1218% (S9), 0.0033% (S12)~0.0437% (S16), and 0.0019% (S18)~0.1398% (S4), respectively. A rough separation of wild and cultivated *Fritillaria* could be achieved by cluster analysis on 23 samples using the content results as cluster variables. 

## 3. Discussion

An approach based on the use UPLC-QTOF-MS was developed to investigate the chemical constituents of wild and artificial cultivated *Fritillaria*, and to conduct a comparison of the results, which was expected to be useful in the foundation of medicinal quality control and material basis research. After analysing the accurate molecular mass and molecular fragments, 89 and 85 components were found in wild and artifical cultivated *Fritillaria*, and 40 alkaloids were elucidated. Based on the experience reported in an earlier study, the product ion at *m*/*z* 114 (C_6_H_12_NO) was formed by the cleavage of the E-ring with the loss of C_21_H_30_O_2_, owing to the presence of an O bridge between the 17 and 23-position, with an electronic induction effect. The product ion at *m*/*z* 414 corresponded to the loss of a d-glucopyranoside unit (162 Da), with further peaks appearing at *m*/*z* 138.12 (C_9_H_16_N), produced by the loss of C_18_H_28_O_2_.

Compound **3** (t_R_ = 0.45 min) produced [M + H]^+^ ions at *m*/*z* 268, and highly abundant fragment ions at *m*/*z* 136 [M + H − 132]^+^ and *m*/*z* 119 [M + H − 149]^+^ in the MS2 experiment, suggesting that adenine was present, and the loss of 149 (132 + 17) Da was attributed to the release of a nucleosides moiety and hydroxyl molecule. According to the previous reports [16,17,18], compound **1** was identified as Adenosine. Compound **12** (t_R_ = 3.68 min) produced [M + H]^+^ ions at *m*/*z* 446. According to the previous reports [20,27], compound **2** was identified as yibeisine. Compound **21** (t_R_ = 6.17 min) produced [M + H]^+^ ions at *m*/*z* 592, and highly abundant fragment ions at *m*/*z* 138 [M + H − 454]^+^ , *m*/*z* 574 [M + H − 18]^+^, *m*/*z* 412 [M + H − 180]^+^, and *m*/*z* 394 [M + H − 198]^+^ in the MS2 experiment, suggesting that gallic acid and the loss of 18 Da could be attributed to the release of a water molecule, and the loss of 180 Da (162 + 18) was attributed to the release of a glucose moiety and water molecule. Moreover, Sterolsc was present. According to the previous reports [20,21], the retention time of the reference standard, compound **21**, was identified as Sipeimine-3β-d-glucoside. Compounds **22**, **24**, **44**, and **59** possessed the same mass weight and fragments as compound **21**, and could be regarded as the isomers of Sipeimine-3β-d-glucoside. 

Compounds **23** and **26** (t_R_ = 6.51 min, 6.99 min) produced [M + H]^+^ ions at *m*/*z* 590, and highly abundant fragment ions at *m*/*z* 428 [M + H − 162]^+^, *m*/*z* 574 [M + H − 16]^+^, *m*/*z* 412 [M + H − 178]^+^, and *m*/*z* 394 [M + H − 196]^+^ in the MS2 experiment, suggesting that the loss of 162 Da was attributed to the release of a glucose moiety, the loss of 16 Da was attributed to the release of an Oxygen atom, and the loss of 196 Da was attributed to the release of Α-d-(+)-glucopyranose. According to the previous reports [22], compounds **23** and **26** were tentatively identified as the isomers of Peimisine-3-*O*-β-d-glucopyranoside. 

Compound **25** (t_R_ = 6.93 min) produced [M + H]^+^ ions at *m*/*z* 594, and highly abundant fragment ions at *m*/*z* 576 [M + H − 18]^+^, *m*/*z* 414 [M + H − 180]^+^, and *m*/*z* 138 [M + H − 456]^+^ in the MS2 experiment, suggesting that the loss of 18 Da was attributed to the release of a water molecule and the loss of 180 Da (162 + 18) was attributed to the release of a glucose moiety and water molecule. According to the previous reports [23], compound **25** was identified as Peiminoside.

Compounds **27** and **28** (t_R_ = 7.15 min, 7.29 min) produced [M + H]^+^ ions at *m*/*z* 444, and highly abundant fragment ions at *m*/*z* 428 [M + H − 16]^+^ , *m*/*z* 114 [M + H − 330]^+^, *m*/*z* 412 [M + H − 32]^+^, and *m*/*z* 142 [M + H − 302]^+^ in the MS2 experiment, suggesting that the release of an oxygen atom was an indicator of the presence of xylose and/or arabinose and a glucose group. According to the previous reports [24], compound **27** was tentatively identified as yibeissine.

Compounds **29**, **39** and **70** (t_R_ = 7.54 min, 9.45 min, 16.3 min) produced [M + H]^+^ ions at *m*/*z* 446. According to the previous reports [6,19], compound **11** was identified as Yibeinine and its isomers. 

Compound **30** (t_R_ = 7.73 min) produced [M + H]^+^ ions at *m*/*z* 430, and highly abundant fragment ions at *m*/*z* 412 [M + H − 18]^+^ and *m*/*z* 138 [M + H − 292]^+^ in the MS2 experiment, suggesting the release of a water molecule and the presence of a glucose group. According to the previous reports [23] and on the basis of the retention time of the reference standard, compound **30** was identified as Sipeimine. Compounds **31**, **32**, **33**, and **36** possessed the same mass weight and fragments as compound **30**, which could be regarded as the isomers of Sipeimine.

Compound **34** (t_R_ = 8.40 min) produced [M + H]^+^ ions at *m*/*z* 432, and highly abundant fragment ions at *m*/*z*414 [M + H − 18]^+^, *m*/*z* 398 [M + H − 34]^+^, and *m*/*z* 138 [M + H − 294]^+^ in the MS2 experiment, suggesting the release of a water molecule, the loss of 18 Da and two ammonia molecules, and the presence of a glucose group. According to the previous reports [23], compound **34** was tentatively identified as Verticine and its isomers.

Compound **35** (t_R_ = 8.77 min) produced [M + H]^+^ ions at *m*/*z* 428, and highly abundant fragment ions at *m*/*z* 412 [M + H − 16]^+^, *m*/*z* 114 [M + H − 314]^+^, and *m*/*z* 142 [M + H − 286]^+^ in the MS2 experiment, suggesting the loss of an oxygen atom, an indicator of the presence of xylose and/or arabinose and a glucose group. Based on the retention time of the reference standard and previous reports [20,23,25], compound **35** was identified as Peimisine. Compound **38** possessed the same mass weight and fragments as compound **35**, which could be regarded as the isomers of Peimisine. 

Compound **41** (t_R_ = 9.74 min, 10.00 min) produced [M + H]^+^ ions at *m*/*z* 592. The product ion at *m*/*z* 574.3744 corresponded to the loss of a H_2_O molecule (−18 Da), with further peaks appearing at *m*/*z* 412 and 394, produced by the loss of a glucose unit (162 Da) and a water molecule (18 Da). According to the previous reports [20], compound **41** was tentatively identified as Yibeinoside A and its isomers.

Compound **54** (t_R_ = 12.89 min), produced [M + H]^+^ ions at *m*/*z* 576. The product ion at *m*/*z* 414, 396, corresponded to the loss of a moiety of glucose and a water molecule. Compared with the retention time of the reference standard, compound **54** was identified as Yibeinoside A. Compounds **41**, **43**, **46**, **47**, **48**, **49**, **50**, and **56** possessed the same fragments and mass weight as compound **54**, and were tentatively identified as the isomers of Yibeinoside A.

Compounds **65** and **69** (t_R_ = 15.66, 16.07 min) produced [M + H]^+^ ions at *m*/*z* 414, and highly abundant fragment ions at *m*/*z* 105 [M + H − 309]^+^ and *m*/*z* 396 [M + H − 18]^+^ in the MS2 experiment, suggesting that gallic acid and a water molecule were released. According to the previous reports [20,23], compound **65** was tentatively identified as puqiedinone and its isomers.

Compounds tentatively identified by the accurate mass weight included compound **66** (t_R_ = 15.75 min), which produced [M + H]^+^ ions at *m*/*z* 608. According to the previous reports [23], compound **66** was identified as puqietinone. Compound **73** was also identified as puqietinone. Compound **78** (t_R_ = 18.09 min) produced [M + H]^+^ ions at *m*/*z* 242. According to the previous reports [18], compound **78** was identified as Thymidine. Compound **86** (t_R_ = 19.89 min) produced [M + H]^+^ ions at *m*/*z* 223. According to the previous reports [26], compound **86** was identified as Patchouli alcohol. The alkaloids were closely connected with the bio-activities in *Fritillaria*. As mentioned above, the alkaloids in *Fritillaria* exhibit a wide range of bioactivities in many aspects, such as anti-micobial, antiumor, antihypertensive, and antiussive activities. In this study, the recognized 40 alkaloids matched well with the biological alkaloids identified in the earlier reports on *Fritillaria* [29], indicating that no wild and cultivated *Fritillaria* possessed the same potential for treating relative diseases due to the same kinds of efficient components.

Four typical alkaloids, namely Sipeimine-3β-d-glucoside, Sipeimine, Peimisine, and Yibeinoside A, were chosen as the standard to build a simple, rapid, accurate, sensible, and reproducible method for the quality control of *Fritillaria*. After analysing the components of *Fritillaria* planted in different places, SPSS was adopted to roughly classify *Fritillaria*, which could be used to screen the substitute of *Fritillaria* with similar components and bioactivities through the identification of the quality and authenticity in *Fritillaria*, providing a reference on the quality evaluation, and playing a significant role in the sufficient utilization and protection of *Fritillaria* medicinal plant resources. 

## 4. Materials and Methods 

### 4.1. Materials and Reagents

A total of 23 samples of *Fritillaria* herbs were collected during 2013 to 2015 from Xinjiang and a herbal medicine market, respectively. They were identified by Sulaiman Halike, a senior pharmacist from the Xinjiang Uygur Autonomous Region Institute for Food and Drug Control (for details, see Table 3). For the species from Kazakhstan, the herbs were collected from the village of Urunkhayka near Lake Markakol in East Kazakhstan. Relative herbs were smashed and dried under a vacuum at 80 ^o^C, and were then sieved (80 mesh) and deposited in an ampubottle before use. The numbers of the herbs were 20130001-1, 20140001-2, and 20150001-6, as stored in the Xinjiang Uygur Autonomous Region Institute for Food and Drug Control. Each sample was crushed, sifted through a 60 mesh sieve, mixed thoroughly, and sealed in a bag, avoiding light. 

Standard substances of Sipeimine-3β-d-glucoside (99.1%, Batch No.110767-201208), Sipeimine (96.4%, Batch No.111917-201202, Beijing, China), and Peimisine (≥98%, Batch No. 111892-201402, Beijing, China) were brought from the National Institute for Food and Drug Control. Standard of Yibeinoside A (>98%, Batch No.141009, Pufei De Biotech Co., Ltd., Chengdu, China) was brought from Chengdu Pufei De Biotech Co., Ltd. Ammonia water (Kelong Chemical Reagent Factory, 20120310, Chengdu, China), chloroform ( Fu Yu Fine Chemical Co., Ltd 20151002, Tianjin, China), and formic acid (Tianjin Kwangfu Fine Chemical Industry Research Institute, 20140629) were all of an analytical grade. Methanol (Fisher, 143714, New York, NY, USA) and acetonitrile (Fisher, 155753, New York, NY, USA) were of a Mass spectrometry grade. The water used was ultrapure water.

### 4.2. Method

#### 4.2.1. Characterization of Chemical Constituents

Liquid Chromatography separation was performed using a Waters ACQUITY UPLC I-Class system (Waters Corporation, Milford, MA, USA) composed of a quaternary pump, an on-line degasser, an autosampler, and a column temperature controller. Then, separations were achieved on a Waters BEH-C18 column (50 mm × 2.1 mm, 1.7 μm) (Waters Corporation, Milford, MA, USA) using an ACQUITYTM Ultra performance Liquid Chromatography system (Waters, Milford, MA, USA). The column temperature was maintained at 40 °C. The mobile phase was a binary mobile phase consisting of 0.1% formic acid-water solution (A) and acetonitrile (B) with an elution gradient of 0~15 min (95%→75% A), 15~22 min (75%→0% A), 22~23 min (0% A), and 23~23.1 min (0%→95% A), while the flow rate was 0.4 mL min^−1^ and the inject volume was 2 μL.

A Waters Xevo G2-S Q-TOF Mass Spectrometer (Waters corporation, Manchester, UK) with an electrospray ionization (ESI) interface was connected with the liquid chromatography. The ESI source was operated in a positive ionization mode with a scanning range of *m*/*z* 50~1200. The ion source parameters were optimized as follows: capillary voltage, 0.5 kV; ion source temperature, 100 °C; desolvation temperature, 450 °C; cone voltage, 40 V; extraction cone voltage, 80 V; cone gas flow, 50 L h^−1^; and solvent-removing flow (N_2_), 800 L h^−1^. Leucine-enkephalin ([M + H]^+^ = 556.2771) was used for real-time correction. All of the operations, data acquisition, and analysis were controlled by MassLynxVer.4.0 software (Waters Corporation, Manchester, UK).

#### 4.2.2. Quantitative Analysis

Chromatographic separation was achieved using a Waters ACQUITY-UPLC BEH-C18 column (100 mm × 2.1 mm, 1.7 μm) (Milford, MA, USA). The column temperature was maintained at 40 °C. The mobile phase was a mixture of 0.1% formic acid-water solution and acetonitrile. Elution gradients of 0~5 min (95%→87% A) and 5~15 min (87%→78% A) were used, while the flow rate was 0.4 mL·min^−1^ and the injected volume was 2 μL. Mass spectrometry was performed in a positive mode with a scanning range of *m*/*z* 50~1200. The ion source parameters were optimized as follows: capillary voltage, 0.5 kV; ion source temperature, 100 °C; desolvation temperature, 450 °C; cone voltage, 40 V; extraction cone voltage, 80 V; cone gas flow, 50 L·h^−1^; and solvent-removing flow (N_2_).

### 4.3. Preparation of Sample Solutions

#### 4.3.1. Characterization of Chemical Constituents

A total of 0.25 g of *Fritillaria* powder was weighed precisely and put into a 50 mL conical flask with a plug, and 25 mL of methanol–water (6:4, *v*/*v*) mixture was then added. The suspension was weighed and extracted under sonication (100 W, 35 kHz) for 30 min, before being cooled, weighed again. It was then added to a methanol–water (6:4, *v*/*v*) mixture to make up for the weight, and was filtered and centrifuged (5000 rpm) for 10 min. The supernatant was collected and dried in an evaporation pan, and the residues were re-dissolved in methanol and transferred to a 5 mL volumetric flask, before methanol was added. The solvent was filtered through a 0.22 μm organic membrane before analysis. 

#### 4.3.2. Quantitative Analysis

In order to conduct an effective qualification of the alkaloids in *Fritillaria*, the selectivity of the compounds was very important. Based on the high resolution of the chromatogram and the high abundance of the content, four compounds, including Sipeimine-3β-d-glucoside, Sipeimine, Peimisine, and Yibeinoside A, were finally selected as a marker for the qualification of alkaloids. Stock solutions of Sipeimine-3β-d-glucoside, Sipeimine, Peimisine, and Yibeinoside A were prepared in methanol at a concentration of 1 mg mL^−1^ and mixed for preparing stock standard solutions for Sipeimine-3β-d-glucoside, Sipeimine, Peimisine, and Yibeinoside A with concentrations of 4.028, 1.015, 4.08, 2.212 μg mL^−1^, respectively. Then, the mixed stock standard solutions were separately diluted with methanol to obtain the standard working solutions. The concentrations of the calibration standard solutions for Sipeimine-3β-d-glucoside, Sipeimine, Peimisine, Yibeinoside A, and Peimisine were 7.867~4028 ng·mL^−1^, 1.982~1015 ng·mL^−1^, 4.320~2212 ng·mL^−1^, and 7.969~4080 ng·mL^−1^, respectively.

A total of 0.25 g of *Fritillaria* powder was precisely weighed and put into a 50 mL conical flask with a plug, before being alkalized with 2 mL ammonia water (25%) for 1 h. Then, 25 mL chloroform–methanol (8:2, *v*/*v*) was added and mixed to perform an ultrasonic extraction (100 W, 35 kHz) for 30 min. After being filtered and dried in an evaporation pan, the residues were re-dissolved in methanol and transferred to a 10 mL volumetric flask, where methanol was added for a constant volume. A total of 1 mL of the solution was transferred into another 10 mL volumetric flask for diluting. The solvent was filtered through 0.22 μm organic membranes before analysis. 

### 4.4. Quantitative Method Validation

A total of 2 μL of different concentrations of the standard working item 4.3.2 was weighed precisely to analyze the chromatographic and mass spectrometry conditions mentioned in Section 4.2.2, and linear regression was carried out between the peak area of the standard sample (Y) and the corresponding concentration, to obtain the regression equation, correlation coefficient, and linearity range. Stepwise dilution was used on the lowest mass concentration of standard reserving liquid mixture. The quantization limit was a signal to noise ratio of 10 and the detection limit was a signal to noise ratio of three. 

### 4.5. Standard Addition Recovery Experiments

The recovery was investigated at low, mediate, and high concentration levels. Sample powders (S8, 0.125 g) were put into a 50 mL conical flask, which was used as the background. Then, the standard solution with a content of the four standards of 80%, 100%, and 120% was added, respectively, and then analyzed under the chromatographic and mass spectrometry conditions mentioned above. The relative results are presented in Table S3.

## 5. Conclusions

An approach based on the use of UPLC-QTOF-MS was developed to investigate the chemical constituents of wild and artificial cultivated *Fritillaria*, and to conduct a comparison of the results, which was expected to be useful in the foundation of medicinal quality control and material basis research. After analysing the accurate molecular mass and molecular fragments, the 89 and 85 components were found in wild and artifical cultivated *Fritillaria*, and 40 alkaloids were elucidated. Four typical alkaloids, namely Sipeimine-3β-d-glucoside, Sipeimine, Peimisine, and Yibeinoside A, were chosen as the standard to build a simple, rapid, accurate, sensible, and reproducible method for the quality control of *Fritillaria*. After analysing the components of *Fritillaria* planted in different places, SPSS was adopted to roughly classify the *Fritillaria* specimens, which could be used to screen a substitute of *Fritillaria* with similar components and bioactivities through the identification of the quality and authenticity of *Fritillaria*, providing a reference on the quality evaluation and playing a significant role in the sufficient utilization and protection of *Fritillaria* medicinal plant resources. 

## Figures and Tables

**Table 1:** Result of chromatographic peak identification of wild *Fritillaria* (Gongliu County, Yili) (S1).

Peak	t_R_/min	Formula	Experiment Value *m/z*	Theroretical Value *m/z*	Error		Fragments	Compound	Relative Mass Fraction %	References	Structural Formula
					mDa	ppm					
1	0.3	C_6_H_14_N_4_O_2_	175.1202	175.1195	0.7	4			0.61		
2	0.35	C_6_H_14_N_4_O_2_	175.1198	175.1195	0.3	1.7			1.47		
3	0.45	C_9_H_17_NO_8_	268.1047	268.1046	0.1	0.4	136.0632, 119.036	Adenosine	0.97	[16,17,18]	
4	0.54	C_12_H_21_NO_6_	276.1445	276.1447	−0.2	−0.7			0.24		
5	0.6	C_10_H_23_NO_6_	254.1617	254.1617	0	0			0.10		
6	0.68	C_9_H_15_NO_4_	202.1081	202.1079	0.2	1			0.09		
7	0.75	C_6_H_9_NO_3_	144.0663	144.0661	0.2	1.4			0.20		
8	0.83	C_8_H_9_N	120.0818	120.0813	0.5	4.2			0.60		
9	0.9	C_15_H_35_NO_10_	390.2355	390.2355	0.1	0.3			0.22		
10	1.45	C_10_H_19_NO_5_	234.1385	234.1341	4.4	3.2			3.17		
11	3.56	C_33_H_51_NO_9_	606.3644	606.3642	1.2	2			0.10		
12	3.68	C_27_H_43_NO_4_	446.3283	446.3284	0.4	0.9		yibeisine	1.65	[6,19]	Figure 2D
13	3.92	C_33_H_51_NO_10_	622.3589	622.3605	−1.6	−2.6			0.09		
14	4.85	C_33_H_51_NO_9_	606.3632	606.3642	−1	−1.6			0.08		
15	4.93	C_33_H_51_NO_9_	606.364	606.3642	−0.2	−0.3			0.05		
16	5.33	C_33_H_51_NO_9_	606.3646	606.3642	0.4	0.7			0.25		
17	5.6	C_33_H_53_NO_9_	608.3805	608.3799	0.5	0.8			0.51		
18	5.73	C_34_H_55_NO_8_	606.4001	606.4006	−0.5	−0.8			0.22		
19	6.02	C_39_H_63_NO_13_	754.4374	754.4378	−1.7	−2.3			0.07		
20	6.08	C_39_H_63_NO_13_	754.438	754.4378	0.2	0.3			0.14		
21	6.17	C_33_H_53_NO_8_	592.385	592.385	0	0	138.1285, 574.3744, 412.3216, 394.3110,	Sipeimine-3β-d-glucoside	0.11	[20,21]	Figure 2A
22	6.35	C_33_H_53_NO_8_	592.4021	592.4015	0.6	1	138.1285, 574.3744, 412.3216, 394.3110	Sipeimine-3β-d-glucoside its isomers	11.95	[20,21]	Figure 2A
23	6.51	C_33_H_51_NO_8_	590.3693	590.3588	0.6	1	428.3167, 412.3211, 114.0921, 142.0782	Peimisine-3-*O*-β-d-glucopyranoside its isomers	4.68	[22]	
24	6.81	C_33_H_53_NO_8_	592.3846	592.3849	−0.3	−0.5	138.1285, 574.3744, 412.3216, 394.3110,	Sipeimine-3β-d-glucoside its isomers	2.53	[20,21]	Figure 2A
25	6.93	C_33_H_55_NO_8_	594.4009	594.4006	0.2	0.3	576.3896, 414.3364, 138.1285	Peiminoside	0.62	[23]	
26	6.99	C_33_H_51_NO_8_	590.3691	590.3693	−0.2	−0.3	428.3167, 412.3211, 114.0921, 142.0782	Peimisine-3-*O*-β-d-glucopyranoside its isomers	0.22	[22]	
27	7.15	C_27_H_41_NO_4_	444.3121	444.3114	0.5	1.1	428.3167, 412.3211, 114.0921, 142.0782	Yibeissine isomers	0.27	[24]	
28	7.29	C_27_H_41_NO_4_	444.3139	444.3127	2.5	5.6	428.3167, 412.3211, 114.0921, 142.0782	Yibeissine isomers	0.13	[24]	
29	7.54	C_27_H_43_NO_4_	446.3274	446.327	0.4	0.9		Yibeinine its isomers	0.21	[6,19]	Figure 2D
30	7.73	C_27_H_43_NO_3_	430.3462	430.3342	2.1	4.9	412.3279, 138.1301	Sipeimine	9.46	[23]	Figure 2B
31	8.00	C_27_H_43_NO_3_	430.3316	430.3321	−0.6	−1.4	412.3279, 138.1301	Sipeimine its isomers	0.07	[23]	Figure 2B
32	8.12	C_27_H_43_NO_3_	430.3313	430.3321	−0.8	−1.9	412.3279, 138.1301	Sipeimine its isomers	0.13	[23]	Figure 2B
33	8.29	C_27_H_43_NO_3_	430.3323	430.3321	0.2	0.5	412.3279, 138.1301	Sipeimine its isomers	1.55	[23]	Figure 2B
34	8.4	C_27_H_45_NO_3_	432.3474	432.3478	−0.4	−0.9	414.3366, 398.3054, 138.1284	Verticine its isomers	0.42	[23]	
35	8.77	C_27_H_41_NO_3_	428.3165	428.3165	0	0	412.3211, 114.0921, 142.0782	Peimisine	4.13	[20,23,25]	Figure 2E
36	9.05	C_27_H_43_NO_3_	430.3383	430.3321	0.1	0.2	412.3279, 138.1301	Sipeimine	3.73	[23]	Figure 2B
37	9.3	C_23_H_33_NO	340.2599	340.2581	0.8	2.4			0.17		
38	9.35	C_27_H_41_NO_3_	428.3165	428.3165	0	0	412.3211, 114.0921, 142.0782	Peimisine its isomers	0.15	[20,23,25]	Figure 2E
39	9.45	C_27_H_43_NO_4_	446.3271	446.3271	0	0		Yibeinine its isomers	0.21	[6,19]	Figure 2D
40	9.62	C_34_H_55_NO_8_	606.4005	606.4006	−0.1	−0.2			0.28		
41	9.74	C_33_H_53_NO_7_	576.3902	576.3901	0.1	0.2	414.3369, 396.3263	Yibeinoside A its isomers	1.01	[25]	Figure 2F
42	9.81	C_33_H_55_NO_7_	578.4049	578.4057	−0.7	−1.2	416.3516, 398.3409	*hupeheninoside*	0.45	[25]	
43	10.00	C_33_H_53_NO_7_	576.39	576.39	0	0	414.3369, 396.3263	Yibeinoside A its isomers	0.08	[25]	Figure 2F
44	10.21	C_33_H_53_NO_8_	592.3848	592.3849	−0.2	−0.3	138.1285, 574.3744, 412.3216, 394.3110	Sipeimine-3β-d-glucoside its isomers	0.30	[20,21]	Figure 2A
45	10.51	C_27_H_41_NO	396.8019	396.8019	0	0			0.25		
46	10.66	C_33_H_53_NO_7_	576.391	576.39	1	1.7	414.3369, 396.3263	Yibeinoside A	6.78	[20]	Figure 2F
47	10.87	C_33_H_53_NO_7_	576.3898	576.39	−0.2	−0.3	414.3369, 396.3263	Yibeinoside A its isomers	1.05	[20]	Figure 2F
48	11.33	C_33_H_53_NO_7_	576.39	576.39	0	0	414.3369, 396.3263	Yibeinoside A its isomers	0.87	[20]	Figure 2F
49	11.6	C_33_H_53_NO_7_	576.396	576.4076	−3.6	−6.2	414.3369, 396.3263	Yibeinoside A its isomers	3.56	[20]	Figure 2F
50	11.81	C_33_H_53_NO_7_	576.396	576.396	0	0	414.3369, 396.3263	Yibeinoside A its isomers	0.12	[20]	Figure 2F
51	11.97	C_28_H_45_NO_2_	428.3163	428.3165	−0.2	−0.5		puqietinedinone its isomers	0.30	[26]	
52	12.14	C_33_H_53_NO_8_	592.3859	592.3849	1	1.7	138.1285, 574.3744, 412.3216, 394.3110,	Sipeimine-3β-d-glucoside its isomers	0.21	[20,21]	Figure 2A
53	12.63	C_27_H_43_NO_2_	414.3461	414.6438	0.9	1.6	396.3253, 105.0697	puqiedinoneits isomers	8.84	[20,23]	Figure 2C
54	12.89	C_33_H_53_NO_7_	576.3909	576.3901	0.9	1.6	414.3369, 396.3263	Yibeinoside A	1.11	[20]	Figure 2F
55	13.1	C_40_H_67_NO_12_	754.4739	754.4742	−0.3	−0.2			0.55		
56	13.19	C_33_H_53_NO_7_	576.3902	576.3901	0.2	0.3	414.3369, 396.3263	Yibeinoside A its isomers	0.93	[20]	Figure 2F
57	13.46	C_27_H_43_NO_2_	414.3411	414.3472	3.9	9.4	396.3253, 105.0697	puqiedinone its isomers	4.03	[20,23]	Figure 2C
58	13.64	C_34_H_57_NO_8_	608.4161	608.4162	−0.1	−0.2		pingbeininoside	0.39	[24]	
59	13.76	C_33_H_53_NO_8_	592.385	592.385	0	0	138.1285, 574.3744, 412.3216, 394.3110	Sipeimine-3β-d-glucoside its isomers	0.51	[20,21]	Figure 2A
60	13.93	C_39_H_65_NO11	724.4642	724.4636	0.6	0.8			1.11		
61	14	C_27_H_43_NO_3_	430.3323	430.3321	0.2	0.5	412.3279, 138.1301	Sipeimine its isomers	0.64	[23]	Figure 2B
62	14.13	C_39_H_65_NO_12_	740.4592	740.4585	0.7	0.9			0.24		
63	15.07	C_27_H_45_NO_2_	416.3525	416.3529	−0.5	−1.2	398.3418	Songbeinine	0.27	[23]	
64	15.13	C_33_H_55_NO_7_	578.406	578.4059	0.1	0.2	416.3516, 398.3409	*hupeheninoside*	0.19	[20]	
65	15.66	C_27_H_43_NO_2_	414.3373	414.3373	0	0	396.3253, 105.0697	puqiedinone its isomers	0.58	[20,23]	Figure 2C
66	15.75	C_28_H_47_NO_2_	430.3636	430.3685	−4.9	−11.4		puqietinone	0.23	[27]	
67	15.82	C_28_H_45_NO_2_	428.3527	428.3529	−0.2	−0.5		puqietinedinone its isomers	0.39	[27]	
68	15.9	C_33_H_55_NO_6_	562.4106	562.4108	−0.2	−0.4			0.49		
69	16.07	C_27_H_43_NO_2_	414.337	414.3372	−0.3	−0.7	396.3253, 105.0697	puqiedinone its isomers	0.63	[20,23]	Figure 2C
70	16.30	C_27_H_43_NO_4_	446.3635	446.3634	0	0		Yibeinine its isomers	0.31	[6,19]	Figure 2D
71	16.37	C_14_H_31_NO_2_	246.2432	246.2433	−0.1	−0.4			0.44		
72	16.58	C_16_H_35_NO_3_	290.2693	290.2695	−0.2	−0.7			0.24		
73	16.79	C_28_H_47_NO_2_	430.3689	430.3685	0.4	0.9		puqietinone	0.35	[27]	
74	17.41	C_13_H_10_O	183.081	183.081	0	0			1.00		
75	17.75	C_16_H_35_NO_2_	274.2798	274.2759	1.3	4.7			3.04		
76	17.82	C_18_H_39_NO_3_	318.3015	318.3008	0.7	2.2			1.50		
77	17.94	C_16_H_33_NO_2_	272.2589	272.259	−0.2	−0.7			0.18		
78	18.09	C_16_H_35_N	242.2849	242.2848	0.1	0.4		Thymidine	0.36	[18]	
79	18.29	C_21_H_30_O_2_	359.2323	359.2324	−0.1	−0.3			0.32		
80	18.76	C_18_H_39_NO_2_	302.3057	302.3059	−0.2	−0.7			0.20		
81	18.86	C_14_H_18_O	203.1437	203.1437	0.1	0.5			0.68		
82	18.96	C_18_H_30_O_2_	279.2321	279.2324	−0.3	−1.1			0.25		
83	19.17	C_17_H_24_O_3_	277.1801	277.1804	−0.3	−1.1			0.37		
84	19.3	C_21_H_37_N	304.3001	304.3004	−0.3	−1			0.20		
85	19.42	C_18_H_20_O_4_	301.1417	301.144	−2.3	−7.6			0.43		
86	19.89	C_15_H_26_O	223.2063	223.2062	0.3	1.3		Patchouli alcohol	0.92	[26]	
87	20.04	C_22_H_32_O_8_	425.2154	425.2175	−1.6	−3.8			0.80		
88	20.55	C_16_H_33_NO	256.264	256.264	0	0			0.18		
89	20.73	C_18_H_35_NO	282.2795	282.2797	−0.6	−2.1			0.26		

**Table 2:** The content of four alkaloids in 23 *Fritillaria* from different sources (%, n = 3).

Number	Sipeimine-3β-d-glucoside	Sipeimine	Peimisine	Yibeinoside A
S1	0.0576	0.0573	0.0155	0.0349
S2	0.0565	0.0395	0.0087	0.0337
S3	0.0084	0.0275	0.0338	0.0236
S4	0.0665	0.0141	0.012	0.1398
S5	0.0044	0.0105	0.0096	0.0034
S6	0.0375	0.0453	0.0096	0.0202
S7	0.0099	0.0216	0.0079	0.0038
S8	0.0338	0.0493	0.0099	0.018
S9	0.0427	0.1218	0.0343	0.0087
S10	0.0749	0.0319	0.0083	0.0357
S11	0.1033	0.0337	0.0073	0.0499
S12	0.0018	0.0113	0.0033	0.0038
S13	0.0815	0.0337	0.0073	0.0558
S14	0.0763	0.0413	0.0126	0.0494
S15	0.0545	0.0471	0.0093	0.0366
S16	0.0039	0.0358	0.0437	0.0132
S17	0.0083	0.0091	0.0113	0.0203
S18	0.0013	0.0066	0.0094	0.0019
S19	0.007	0.0152	0.0124	0.0051
S20	0.0737	0.1086	0.0071	0.008
S21	0.0882	0.0913	0.0058	0.0076
S22	0.0088	0.0755	0.0206	0.0075
S23	0.1357	0.1019	0.0067	0.0116

**Table 3:** *Fritillaria* source.

Sampel No.	Provenance	Origin	
S1	(Wild)	*F. pallidiflora*	Gongliu County, Yili
S2	(Cultivate)	*F. pallidiflora*	Gongliu County, Yili
S3	(Wild)	*F. walujewii*	Xinyuan County, Yili
S4	(Wild)	*F. pallidiflora*	Huocheng County, Yili
S5	(Wild)	*F. tortifolia*	Toli County, Tacheng
S6	(Cultivate)	*F. yuminensis*	Yumin County, Tacheng
S7	(Wild)	*F. yuminensis*	Yumin County, Tacheng
S8	(Cultivate)	*F. tortifolia*	Toli County, Tacheng
S9	(Cultivate)	*F. walujeweii*	Altai, Xinjiang
S10	(Wild)	*F. pallidiflora*	Tekes County, Yili
S11	(Wild)	*F. pallidiflora*	Tekes County, Yili
S12	(Cultivate)	*Fritillaria*	Kazakhstan
S13	(Cultivate)	*F. pallidiflora*	Yili, Xinjiang
S14	(Cultivate)	*F. pallidiflora*	Yili, Xinjiang
S15	(Wild)	*F. pallidiflora*	Yili, Xinjiang
S16	(Wild)	*F. walujeweii*	Altai, Xinjiang
S17	(Wild)	*F. verticillata*	Jeminay County,Altai
S18	(Wild)	*F. tortifolia*	Toli County, Tacheng
S19	(Wild)	*F. yuminensis*	Yumin County, Tacheng
S20	(Wild)	*Fritillaria*	Kazakhstan
S21	(Wild)	*Fritillaria*	Kazakhstan
S22	(Wild)	*F. yuminensis*	Yumin County, Tacheng
S23	(Wild)	*Fritillaria*	Kazakhstan

## Supplementary Materials

The following are available online, Table S1: Regression equation, LODs and LOQs of quantitative method for four alkaloid, Table S2: The results of precision, stability and repetition experiment, Table S3: The results of recovery experiments.
