# Supplementary material for: Rapid Quantification and Quantitation of Alkaloids in Xinjiang Fritillaria by Ultra Performance Liquid Chromatography-Quadrupole Time-of-Flight Mass Spectrometry
Source: Molecules. 2017 May 1;22(5):719. doi: 10.3390/molecules22050719 (PMC6154541; doi:10.3390/molecules22050719)
Supplement: Supplementary file 1 [file molecules-22-00719-s001.pdf]

Table S1 Regression equation, LODs and LOQs of quantitative method for four alkaloid

compound	linear range(ng/mL)	regression equation	R	LODs(ng/mL)	LOQs(ng/mL)
Sipeimine-3*β*-D-glucoside	7.867~4028	Y = 181.8X + 4129.5	0.9994	0.1229	0.2458
Sipeimine	1.982~1015	Y= 375.08X + 4374.8	0.9991	0.2478	0.4956
Peimisine	4.320~2212	Y = 329.69X + 6301.7	0.9993	0.1350	0.5400
Yibeinoside A	7.969~4080	Y = 147.32X + 304.87	0.9998	0.06226	0.2490

Table S2 The results of precision, stability and repetition experiment

Compound	Precision degree (n=6)		Repeatability (n=6)		Stability (n=5)	
	content(%)	RSD(%)	content (%)	RSD(%)	content (%)	RSD(%)
Sipeimine-3*β*-D-glucoside	0.0338	4.89	0.0316	6.53	0.0328	6.28
Sipeimine	0.0509	1.26	0.0475	5.26	0.0501	3.05
Peimisine	0.0098	1.37	0.0095	5.14	0.0100	2.09
Yibeinoside A	0.0176	2.386	0.0169	6.95	0.0181	2.63

Table S3 The results of recovery experiments

Component	Orginal (μg)	Added (μg)	Measured (μg)	Recovery (%)	Average recovery (%)	RSD (%)
Sipeimine-3*β*-D-glucoside	40.95	25.16	67.04	103.70	101.8	1.95
		40.28	81.99	101.89		
		55.38	96.18	99.73		
Sipeimine	61.02	35.52	95.60	97.35	103.5	5.15
		60.90	125.58	106.01		
		86.28	153.39	107.06		
Peimisine	11.32	8.30	19.90	103.37	100.7	2.82
		11.06	22.48	100.90		
		16.59	27.53	97.71		
Yibeinoside A	22.41	15.30	37.80	100.59	99.80	0.82
		20.40	42.78	99.85		
		30.60	52.69	98.95		
